# Identification and Characterization of Perennial Ryegrass (*Lolium perenne*) Vernalization Genes

**DOI:** 10.3389/fpls.2021.640324

**Published:** 2021-03-05

**Authors:** Rowan Herridge, Samarth, Lynette Brownfield, Richard Macknight

**Affiliations:** Department of Biochemistry, University of Otago, Dunedin, New Zealand

**Keywords:** flowering time, vernalization, grasses, forage, transcriptome

## Abstract

Perennial ryegrass (*Lolium perenne*) is a temperate grass species commonly used as pasture for livestock. Flowering (heading) of ryegrass impacts metabolizable energy content and seed yield, therefore this trait is important for both farmers and seed producers. In related grass species, the *VRN* genes (*VRN1*-*3*) have been largely implicated in the determination of vernalization response and are responsible for much of the intra-species variation in this trait. Many other important flowering-time regulators have been cataloged in the model grass *Brachypodium distachyon;* however, in several cases, such as *VRN2*, their ryegrass homologs have not been well-characterized. Here, ryegrass homologs of important flowering time genes from *B. distachyon* were identified through available synteny data and sequence similarity. Phylogenetic analysis of *VRN3/FT-like* and *VRN2-like* genes was performed to elucidate these families further. The expression patterns of these genes were assessed during vernalization. This confirmed the key roles played by *LpVRN1* and *LpFT3* in the promotion of flowering. Furthermore, two orthologs of *VRN2* identified here, as well as an ortholog of *CO9*, were expressed prior to vernalization, and were repressed in flowering plants, suggesting a role in floral repression. Significant variability in expression of these flowering pathway genes in diverse genotypes was detected and may underlie variation in flowering time and vernalization response.

## Introduction

Perennial ryegrass (*Lolium perenne*) is a temperate grass, widely grown as pasture for livestock for its high levels of metabolizable energy. Ryegrass flowering (heading) occurs in the spring after a period of cold (i.e., winter) referred to as vernalization. As ryegrass is an obligate outcrosser, it is important that minimal variation in heading date occurs within a cultivar to ensure good seed production. Heading also impacts the availability of metabolizable energy, as plants switch from producing leaves toward less-nutritious stems and floral structures (Stockdale, 1999). Selection for different heading dates occurs during the breeding process, and many varieties with early and late heading dates are currently available. While some variation in heading date has been captured through introgression, a better understanding of the genetic mechanisms underlying heading date will enable breeders to develop cultivars with more specific and reliable heading dates.

The genetic mechanisms of flowering have been well studied in other model plants, such as *Arabidopsis thaliana* (Arabidopsis) and *Brachypodium distachyon*. In addition, a number of important flowering time genes have been identified in related crops such as wheat (*Triticum* sp.), barley (*Hordeum vulgare*) and rice (*Oryza sativa*). This intensive study has led to the description of a number of important genes and pathways controlling flowering time in grasses. The genes controlling vernalization response have been particularly well studied, as natural variation in vernalization requirement is widespread amongst different cultivars of wheat and barley. VRN1 is a MADS-box transcription factor which is induced by low temperatures (Yan et al., 2003). Extended periods of low temperatures result in mitotically stable expression of *VRN1* through epigenetic marks (Oliver et al., 2009). Some varieties of spring wheat and barley have mutations at the *VRN1* locus which result in constitutive expression of *VRN1* and do not require vernalization in order to flower (Yan et al., 2003; Chu et al., 2011). Upon transition to long days, *VRN1* is capable of activating transcription of the *FLOWERING LOCUS T* (*FT*) gene, *VRN3*, in leaves (Shimada et al., 2009). The VRN3/FT protein is transported from the leaves to the apical meristem, where it acts as a transcriptional co-activator (Tamaki et al., 2007). *VRN1* is induced by FT in concert with a number of other proteins in the meristem, resulting in transcriptional activation of genes involved in floral meristem production (Li and Dubcovsky, 2008). In barley and *B. distachyon* natural variation exists in a relative of *FT*, *HvFT3*/*BdFTL9*, which acts during short days to promote flowering (Faure et al., 2007; Qin et al., 2019; Woods et al., 2019). In the case of *BdFTL9*, this is through a process called short day vernalization, and promotes floral competency upon return to long days (Woods et al., 2019).

Prior to vernalization, *VRN2* is expressed in leaves, which represses *VRN3* and possibly *VRN1* (Yan et al., 2004; Dubcovsky et al., 2006). VRN2 is a zinc-finger CCT-domain protein and is down-regulated by short days and cold through the activity of VRN1 at the promoter (Chen and Dubcovsky, 2012; Deng et al., 2015). The CCT-domain is named after a 43-amino acid C-terminal domain found in three Arabidopsis proteins, CONSTANS (CO), CO-LIKE, and TIMING OF CAB1 (TOC1). Variations in the CCT domain of VRN2 have a profound impact on vernalization response in wheat, with non-functional variants abolishing vernalization requirement in spring varieties (Distelfeld et al., 2009). In rice, the CCT-domain protein Ghd7 acts to repress flowering in long days and is the ortholog of *VRN2* from wheat (Griffiths et al., 2003; Xue et al., 2008; Woods et al., 2016). Other close homologs of *VRN2/Ghd7* include the *CO9-like* genes, although these are less well-studied (Woods et al., 2016). The *CO9-like* gene from barley is expressed in short-day conditions and acts to repress flowering, suggesting that *CO9-like* genes also act as floral repressors (Kikuchi et al., 2012).

Ryegrass has been the subject of many flowering time studies, primarily using quantitative trait analysis, and a number of important flowering time genes have been identified (reviewed by Wang and Forster, 2017). *VRN1* has been identified in ryegrass, and natural variation in the first intron affects vernalization response (Jensen et al., 2005; Andersen et al., 2006; Asp et al., 2011). A number of *FT-like* genes have also been described in ryegrass, with some of these homologs in close proximity to QTL affecting flowering time (Studer et al., 2009). Allelic variation at *FT* genes underlying these QTL has not yet been described. However, variation at the promoter of *LpFT3*, a putative ortholog of *FT*/*VRN3* genes from other grasses, has been proposed to impact flowering time in a panel of different ryegrass germplasms (Skøt et al., 2011; Wang and Forster, 2017). No ortholog of *VRN2* has been thoroughly described in ryegrass, although a locus on LG4 and a partial sequence have been reported (Paina et al., 2016; Woods et al., 2016). Two genes, named *vrn2_2* and *vrn2_3* were identified in a QTL analysis and corresponded to two CCT-domain proteins on LG7 (Andersen et al., 2006). *Vrn2_2* was reported to be similar to *VRN2* from wheat, while *Vrn2_3* was a *CO* homolog (*LpCO*), although neither gene appeared to be regulated by vernalization.

Recently, a number of genomic resources have been developed for ryegrass, including two draft genomes, transcriptomic data, synteny data, and high-density mapping data (Studer et al., 2012; Paina et al., 2014; Byrne et al., 2015; Velmurugan et al., 2016). In this study, we aimed to use these resources to identify orthologs of important flowering time genes from *B. distachyon*. We further investigated *FT-like* genes and CCT-domain genes in an effort to find orthologous genes to *BdFTL9*, *BdVRN3* and *BdVRN2*. We characterized homologs of *VRN2* in a commercial cultivar during vernalization, flowering, and post-flowering. Finally, we looked for variation in the expression of these genes in a variety of different genotypes, which may underlie differences in their flowering habit.

## Materials and Methods

### Plant Materials and Growth Conditions

*Lolium perenne* plants were grown at 22°C 16 h light/8 h dark (Long Days; LD) or 8 h light/16 h dark (Short Days; SD) unless otherwise stated. A list of the ryegrass cultivars and accessions used in this study is provided in Table 1.

**TABLE 1:** Details of different genotypes used in this study.

Name/Origin/MFGC^a^ ID	Heading date^b^
Nui (Grasslands, New Zealand)	Mid (+ 0)
“Cyprus” (A15336/PI206376)	Early^c^
“Turkey” (B5186/PI173724)	Early
Medea (Australia/Algeria)	Early
“Iran” (A15352/PI227020)	Early^c^
Barberia (Barenbrug, New Zealand)	Early^c^ (−21)
Tyson (Barenbrug, New Zealand)	Early (−10)
Tabu (Barenbrug, New Zealand)	Late (+10)^c^
Alto (Barenbrug, New Zealand)	Late (+14)
Trojan (Barenbrug, New Zealand)	Late (+16)
Kleppe (Norway)	Late^d^
“Sweden” (A15365/PI265335)	Late^d^
“Japan” (A24819/PI420127)	Late^d^
“Norway” (A171783/PI577269)	Late^d^
ONE50 (PGG Wrightson, New Zealand)	Late (+20)

*^*a*^Margot Forde Germplasm Centre. ^*b*^Heading date relative to Nui in brackets (data from supplier). ^*c*^No vernalization requirement. ^*d*^Strong vernalization requirement.*

### Flowering Time Gene Identification

tBLASTn was performed using *B. distachyon* peptides from *B. distachyon* predicted proteins (v1.2) against the *L. perenne* genome (The International Brachypodium Initiative, 2010; Byrne et al., 2015). For ease of identifying the matching annotations, predicted proteins from the ryegrass genome (Byrne et al., 2015) were also searched (BLASTp), and in cases where the top tBLASTn hit matched the top annotated protein hit, the annotation was noted and classified as the most-likely homolog. In cases where these did not match, the tBLASTn hit was examined on a genome browser (Integrative Genomics Viewer, IGV) to identify overlapping masked transcripts, or unannotated regions. The sequences of these genes/regions were aligned to their *B. distachyon* homologs to identify exons, and RNAseq reads (Paina et al., 2014) were simultaneously examined to determine splice sites where possible.

### Phylogenetic Analysis of FT-Family and CCT-Domain Proteins

Homologous FT-family protein sequences in ryegrass were obtained from Veeckman et al. (2016). Homologous FT-like genes from *B. distachyon*, *O. sativa* and *H. vulgare* were also identified from the literature (Griffiths et al., 2003; Higgins et al., 2010). CCT-protein sequences were extracted from the *L. perenne* draft genome by searching predicted proteins (BLASTp) and a translated nucleotide database (tBLASTn) with the CCT domain of *B. distachyon* VRN2 (Byrne et al., 2015). Annotated CCT-domain proteins were identified in *B. distachyon*, *O. sativa* and *H. vulgare* genomes by searching for PFAM domain 06203 on Phytozome (*B. distachyon* and *H. vulgare*)^1^, or the rice genome^2^. All sequences were aligned using MUSCLE program followed by a gblock scan to identify conserved regions among the alignment which can later be used to infer phylogenetic relationship (Edgar, 2004). Phylogenetic trees were created using the CLC Genomics workbench (version 10) with the following settings: maximum likelihood method with JTT substitution matrix and 1000 bootstrap replication.

### RNAseq Data Analysis

Previously published RNAseq data from two ecotypes (Falster and Veyo) was re-analzsed. Full details of the experimental conditions can be found in Paina et al. (2014). To summarize, leaf tissue from clonal Falster and Veyo plants was taken in short day conditions prior to vernalization in short days at 5°C. Leaf tissue was sampled after 2 days, 4 weeks, and 9 weeks of vernalization, and 7 days after returning to long days. Meristem tissue was taken after 9 weeks of vernalization, and 1 day, and 7 days after returning to long days. Raw data from Paina et al. (2014; EMBL-EBI ArrayExpress, accession no. E-MTAB-2623) was mapped to the *L. perenne* genome (Byrne et al., 2015) using STAR mapper (Dobin et al., 2013). Feature counts were extracted using HT-seq count (Anders et al., 2015) with the following settings: htseq-count -q -s no -f bam -t gene -i ID. A customized.gff file containing only the flowering genes of interest was used to avoid overlapping features preventing accurate quantification. Expression levels of each gene were normalized by the total number of mapped reads, and by the transcript length for plotting.

### Sample Collection

For the 10 week vernalization time course, seeds of ONE50 were sterilized in a 1% bleach solution for 15 min and placed onto moist sterile filter paper in a petri dish and sealed with cling film. Seeds were chilled for 2 days in the dark at 4°C before transfer to LD conditions to germinate for 2 weeks, at which point seedlings are typically capable of responding to vernalization (Gangi et al., 1983). After 2 weeks in LD conditions, plates were transferred to 4°C in the dark for 10 weeks, and after 10 weeks of vernalization, seedlings were transferred to soil and grown in LD conditions at 22°C. Samples were collected prior to vernalization (0dV) and at 1 day, 1 week, 2 weeks, 4 weeks, 7 weeks, and 10 weeks vernalization (1dV, 1wV, 4wV, 7wV and 10wV), and at 7 days, 2 weeks and 4 weeks post-vernalization (7dLD, 2wLD, 4wLD). Each RNA sample from 0dV until 10wV was prepared by pooling tissue from the primary leaf of ∼10 seedlings. Samples after transfer to soil were prepared from a pool of single leaves from 3 plants. Samples were taken between 1–2 h after lights were turned on (ZT1- ZT2; 9am–10am), and at the same time each day in dark conditions. Leaf tissue was frozen in liquid nitrogen prior to RNA extraction.

For the comparison of flowering and non-flowering plants, each sample was prepared from leaves from several tillers from a single plant. Leaves were taken from three flowering and three non-flowering plants at ∼6 weeks after the transition to LD conditions when spikelets had clearly emerged in flowering plants. Further samples were taken from these plants 8 weeks later, and after 1 day or 1 week under vernalization conditions (SD + 5°C).

For comparison of gene expression between genotypes, seeds of the cultivars/accessions shown in Table 1 were sterilized and germinated as above for ONE50. Samples were taken after 2–3 weeks growth in long days (0dV) and after 2 days of cold (4°C)/dark treatment (2dV). Each RNA sample was prepared by pooling tissue from the primary leaf of ∼10 seedlings. Samples were taken between 1–2 h after lights were turned on (ZT1- ZT2; 9am–10am), and at the same time each day in dark conditions.

For analysis of *LpFT08* expression in SD conditions, ONE50 plants were grown for 8 weeks in LD conditions (20 h light; 4 h dark). Leaf material from each plant was collected and pooled at T0 (prior to SD treatment), and 4 plants were moved to SD (8h light; 16 h dark) and 4 plants remained in LD (20 h light; 4 h dark). After 2.5 weeks in SD or LD, several leaves from each plant were pooled into a single sample (SD or LD) and frozen in liquid nitrogen prior to RNA extraction.

### RNA Extraction, cDNA Synthesis and qPCR

Frozen leaf tissue (∼10 mg) was thawed in 300 μL Trizol (Invitrogen) prior to transfer to a ziplock bag. A dowel was rolled over the bag to homogenize the leaf tissue. Debris was removed by centrifugation at 16,000 × *g* for 5 min and discarding the pellet. RNA was extracted using Zymo Quickzol RNA microprep kit according to manufacturer’s instructions, eluting in 15 μL of water. cDNA was synthesized with 1 μg of RNA using SuperScript III Reverse transcriptase (Invitrogen) and oligo dT primers according to manufacturer’s instructions. qPCR was performed with SYBR 2x Mastermix (KAPA), using 0.2 μM each primer, in a final volume of 10 μL. Reactions were run in a Roche Lightcycler 480 with the following cycling conditions: a hold of 94 °C for 2 min followed by 50 cycles of 94 °C for 8 s, 58 °C for 10 s and 72 °C for 10 s. Tumor Homolog Protein (*THP*; Samarth and Jameson, 2019) and “expressed protein” (*ExP*; Narsai et al., 2010) were used as reference genes, and gene expression was calculated using the ΔCt method based on the geometric mean Ct of the two reference genes. Primers used for each gene are given in Supplementary Table 1. To compare variance of gene expression between different target genes in the array of genotypes, expression level of each sample was first normalized to the mean value, followed by pairwise F-tests to compare the variance of each target gene (*LpVRN1*, *LpVRN2a/b* and *LpCO9*). The log_2_ fold-change in expression level upon introduction to vernalization conditions was also calculated, and subsequent pairwise F-tests were performed on these values to determine significantly variable responses between target genes.

## Results

### Identification of Flowering Time Gene Homologs Based on Synteny and Sequence Similarity

To investigate the roles of known flowering time genes in vernalization response, we firstly identified likely orthologs of important flowering time genes from related grass species. A list of important flowering time proteins in *B. distachyon* identified by Higgins et al. (2010) was used to search for ryegrass homologs by searching a translated nucleotide database (tBLASTn) and this analysis was supplemented by identifying putative genomic locations using the *L. perenne* GenomeZipper (Byrne et al., 2015). In most cases, regions/genes identified in the GenomeZipper as homologous to *B. distachyon* flowering genes also contained the top tBLASTn hit. In some cases, no syntenic regions were identified in the GenomeZipper, and in these cases, the top BLAST hit was chosen as the most likely ortholog. In cases where the top BLAST hit was already associated with another *B. distachyon* gene, preference was given to the gene that was predicted to be syntenic (i.e., present in the GenomeZipper), and the non-syntenic gene was left without a likely homolog. Overall, from 158 *B. distachyon* genes, 89 had ryegrass homologs present in the GenomeZipper, while 56 had likely homologs identified via BLAST search, and 13 had no confidently predicted homologs. Five tBLASTn hits occurred in regions without an annotated transcript, in which case a note of the location of the BLAST hit was recorded, and the predicted protein was identified for future use (Supplementary Datasets 1, 2). A number of annotated ryegrass genes were previously omitted from the transcriptome/proteome (Byrne et al., 2015); the protein sequences of these genes have also been predicted and included in Supplementary Dataset 2. We also identified a small number of genes with truncated sequences in the proteome, which we manually re-annotated using homology to *B. distachyon* and RNAseq data to predict intron/exon boundaries and identify likely start and stop codons (Supplementary Dataset 2; Paina et al., 2014).

To validate these genes as orthologs of their respective *B. distachyon* genes, we performed a reciprocal BLAST search using their protein sequences against the *B. distachyon* proteome. Of the 89 homologs identified in the GenomeZipper, 82 were also the top hit to their respective *B. distachyon* counterpart, suggesting they are orthologous (Supplementary Dataset 1). A number of these genes have been previously identified via QTL analyses or other means, such as *LpVRN1, LpCO* and *LpVRN3/FT3*. Of particular interest are orthologs of important flowering time genes that have not been previously described. We discovered likely orthologs of *B. distachyon VRN2* (discussed later), *LEAFY* (on scaffold_1714), and *BdSOC1* and *BdSOC1-like* (on scaffold_156 and scaffold_10825). The expression of all identified homologs/orthologs during vernalization was assessed using available RNAseq data from leaves and meristems (Paina et al., 2014; Supplementary Dataset 3). This data was generated by sampling two genotypes (Falster and Veyo) with differing vernalization requirements at several time points during and after vernalization. Combining the expression pattern data for each gene, alongside its homology to known *B. distachyon* genes, unique identifiers from the draft genome, and putative genomic location, allows researchers to rapidly access useful information about these genes without the need to re-analyze next generation sequencing data.

### Phylogeny and Expression Patterns Indicate *LpFT3* and *LpFT08* Likely Play Key Roles in Regulation of Ryegrass Flowering

**FIGURE 1:**
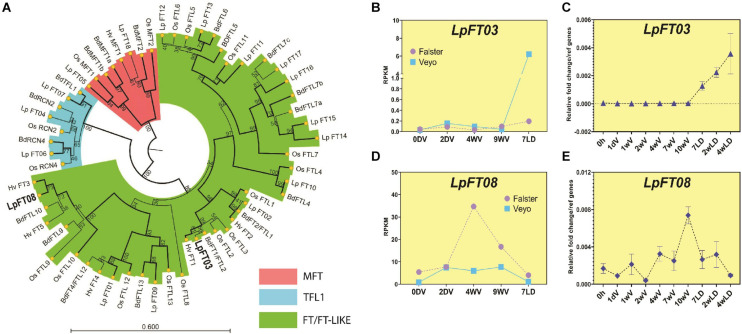
Phylogenetic relationships and expression patterns of FT-like proteins/genes from *L. perenne*. **(A)** Phylogenetic tree of LpFT01-LpFT18 proteins and their homologs from *B. distachyon* (Bd), *H. vulgare* (Hv) and *O. sativa*. (Os). The evolutionary history was inferred by using the Maximum Likelihood method based on the JTT matrix-based model. The bootstrap consensus tree inferred from 1000 replicates is taken to represent the evolutionary history of the taxa analyzed (scores represent the bootstrap support as a percentage). Branches with bootstrap support >70 are shown in bold. Branches in red represent the MFT clade, blue represent the TFL1 clade and green represent the FT clade. Bold highlights genes analyzed further in this study. MFT is the ancestral clade to TFL and FT clades and acts as an outgroup to root the tree. **(B)** Expression pattern of *LpFT3* during and after vernalization in Falster and Veyo cultivars from RNAseq (data from Paina et al., 2014). **(C)** Expression pattern of *LpFT3* during and after vernalization using qPCR in seedlings of the ONE50 cultivar. Expression was normalized to the geometric mean of two reference genes (*ExP* and *THP*), and shows the mean and standard deviation of two biological replicates of leaf tissue from ∼10 pooled seedlings. **(D)** Expression of *LpFT08* during and after vernalization in Falster and Veyo from RNAseq (data from Paina et al., 2014). **(E)** Expression pattern of *LpFT08* during and after vernalization using qPCR in seedlings of the ONE50 cultivar. Expression was normalized to the geometric mean of two reference genes (*ExP* and *THP*) and shows the mean and standard deviation of two biological replicates of leaf tissue from ∼10 pooled seedlings. RPKM, Reads Per Kilobase of transcript per Million mapped reads.

*FT-like* genes are responsible for many aspects of flowering, and duplications and neo-functionalization of genes often occur in this family (Pin and Nilsson, 2012). For this reason, it is possible that there are some duplications or deletions of *FT-like* genes in *L. perenn*e that confound the homology search performed above. For example, *BdFTL7a* and *BdFTL7b* both matched the same ryegrass gene using tBLASTn, which may indicate a duplication in *B. distachyon* or a deletion in *L. perenne*, and therefore requires further analysis. *FT-like* genes have been previously identified in ryegrass which we matched with annotated transcripts from the ryegrass proteome/transcriptome (Byrne et al., 2015; Veeckman et al., 2016; Supplementary Dataset 4). We constructed a phylogenetic tree of these *FT* genes, alongside those from *B. distachyon*, rice, and barley (Figure 1A). This analysis revealed that *LpFT3* was most likely to be the ortholog of floral-inducers (e.g., *BdFTL2*/*BdFT1* and *OsFTL2* aka *Hd3a*; bootstrap support, 99) as has been previously proposed, and this was consistent with the synteny analysis, where it was predicted as the most likely ortholog of *BdFTL2* (Supplementary Dataset 1). *B. distachyon FTL9* has been implicated in a process known as short day vernalization, and grouped with *LpFT08*, as well as *HvFT3* and *BdFTL10* (Figure 1A; bootstrap support, 100; Qin et al., 2019; Woods et al., 2019). A ryegrass ortholog of *TERMINAL FLOWER 1* (*TFL1*) has been previously identified, *LpTFL1*, which was closely grouped with *BdTFL1* (bootstrap support, 91; Jensen et al., 2001). *RCN2* and *RCN4* are also important members of the FT family, controlling grain production in rice, and orthologs of these were detected in ryegrass – *LpFT04* and *LpFT06*, respectively (bootstrap support, 68 and 46; Nakagawa et al., 2002; Ariyaratne et al., 2009). Despite the low bootstrap values of *LpFT04* and *LpFT06*, these were also reciprocal BLAST hits of their *B. distachyon* orthologs (*BdRCN2* and *BdRCN4*), although more comprehensive analysis may be required to fully elucidate the *RCN2*/*RCN4* clade (Supplementary Dataset 1). In *B. distachyon*, *FTL7* has three copies (*BdFTL7a, b* and *c*), while ryegrass has four copies (*LpFT14-17*; bootstrap support, 96), although these genes are yet to be characterized. Ryegrass also had two copies of *MFT*, one closer to *BdMFT1a/b* and one closer to *BdMFT2* (*LpFT05* and *LpFT18*, respectively; bootstrap support, 99 and 99).

To determine which of the *FT-like* genes from ryegrass were responding to vernalization, we examined levels of *FT-like* genes during vernalization using available RNA-seq data (Paina et al., 2014). As expected of a floral inducer, *LpFT3* expression occurred after vernalization when plants were returned to long days (Figure 1B). No other *FT-like* gene showed a similar expression pattern, suggesting that *LpFT3* is likely to be the predominant *FT-like* gene acting as a floral inducer in these conditions (Supplementary Dataset 4). We confirmed the expression pattern of *LpFT3* in a commercial cultivar (ONE50) during vernalization, which was similar to RNA seq data, despite differences in experimental approach (Figure 1C; see Methods). *LpFT3* was also more highly expressed in flowering plants than non-flowering plants resulting from this experiment (discussed later; Supplementary Figure 1A). RNAseq data showed that *LpFT08* was expressed during vernalization, and repressed upon introduction to long days, suggesting that it may act in a similar manner to *BdFTL9* which is activated during short days (Figure 1D). *LpFT08* expression in our vernalization conditions was not similar to the RNAseq data, as it initially decreased in expression, before increasing with prolonged vernalization (Figure 1E). This may be caused by differences in the genotype used for this study, or the difference in vernalization treatments. As the vernalization treatment in this experiment occurred under dark conditions, we tested *LpFT08* expression in short days, which are known to induce the expression of *BdFTL9* (Woods et al., 2019). *LpFT08* expression increased in short days in the ONE50 cultivar in the absence of cold treatment, similarly to *BdFTL9* (Supplementary Figure 1B). Initially we classified *LpFT08* as an ortholog of *BdFTL9* as these genes were linked in the GenomeZipper (Supplementary Dataset 1). However, *LpFT08* grouped with *BdFTL9* and *BdFTL10* in the phylogenetic tree (Figure 1A) and we observed that *BdFTL10* was absent from the GenomeZipper. To determine if *LpFT08* was syntenic to *BdFTL10* we examined the surrounding genes on scaffold_5827 and *B. distachyon* chromosome 2, revealing synteny between *LpFT08* and *BdFTL10* (Supplementary Figure 1C). *LpFT08* is likely to be present on LG1 between markers PTA.648.C1 and P5G13, as that is the putative location of other genes on scaffold_5827.

Overall, the phylogenetic analysis of ryegrass *FT-like* genes has confirmed the identification of *LpFT3* as a likely floral inducer, and *LpFT08* as a potential inducer of floral competency similar to *BdFTL9*. We also grouped other known *FT-like* genes (such as *LpTFL1*) with their appropriate *B. distachyon* orthologs, and this has matched with the synteny data above. This suggests that our approaches to finding important flowering-time genes are appropriate and may be applied to less-well-known gene families.

### Phylogenetic Analysis of CCT-Domain Proteins Revealed Candidates for *VRN2* and *CO9* in Ryegrass

**FIGURE 2:**
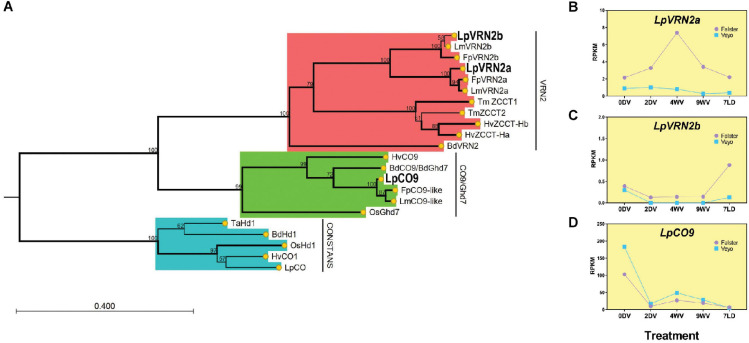
Phylogenetic relationships and expression patterns of VRN2/CO9-like proteins/genes from *L. perenne*. **(A)** Phylogenetic tree containing VRN2-like and CO9-like proteins from *L. perenne* (Lp), *L. multiflorum* (Lm), *F. pratensis* (Fp), *B. distachyon* (Bd), *O. sativa* (Os), *T. monococcum* (Tm), and *H. vulgare* (Hv). The evolutionary history was inferred by using the Maximum Likelihood method based on the JTT + G matrix-based model. The bootstrap consensus tree inferred from 1000 replicates is taken to represent the evolutionary history of the taxa analyzed (scores represent the bootstrap support as a percentage). Branches with bootstrap support >70 are shown in bold. Branches in red represent the VRN2 clade, green represent the CO9/Ghd7 clade and blue represent the CONSTANS clade. CONSTANS acts as an outgroup to root the tree. **(B–D)** Expression patterns of *LpVRN2a*, *LpVRN2b* and *LpCO9* during and after vernalization in Falster and Veyo cultivars from RNAseq (data from Paina et al., 2014). Expression was normalized to total mapped reads and transcript length. RPKM, Reads Per Kilobase of transcript per Million mapped reads.

To characterize the CCT-domain encoding genes from ryegrass, we queried predicted peptides from a translated nucleotide database based on the draft genome of *L. perenne* using the 43 amino acid CCT domain from BdVRN2 (Bradi3g10010) with tBLASTn. Simultaneously, we queried predicted proteins from the ryegrass transcriptome, to account for some cases where an intron interrupts the CCT domain (Cockram et al., 2012; Byrne et al., 2015). We identified annotated transcripts for each hit from which we derived the protein sequences (Supplementary Dataset 5). We also included the “*vrn2_2*” gene (present on scaffold_1808) which has been previously described but was not detected in our tBLASTn search as the CCT domain spans an intron, and is absent from the transcriptome (Andersen et al., 2006). Thirty-six CCT-domain proteins from ryegrass were aligned with CCT-domain proteins from *B. distachyon*, rice, and barley to generate a phylogenetic tree (Supplementary Figure 2). The ryegrass genes fell broadly into clades including *PPD-like*, *CONSTANS-like*, *VRN2/Ghd7/CO9*. From this tree we identified two putative *VRN2-like* genes, as well as a *CO9-like* gene in ryegrass. The gene annotated as “*vrn2_2*” present on scaffold_1808 was more similar to *CO-like 14* from *B. distachyon* (Supplementary Figure 2; Andersen et al., 2006; Cockram et al., 2012). As no ryegrass homolog of *VRN2* has been fully described to-date, we examined the *VRN2*/*Ghd7*/*CO9-like* clade more closely to identify genes which may act as floral repressors in a manner similar to *VRN2* from wheat and barley (Figure 2A). From this tree, we could detect two possible homologs of *VRN2* in ryegrass, present on scaffold_936 (*LpVRN2a*) and scaffold_1700 (*LpVRN2b*). These two genes had homologs in the *L. multiflorum* and *F. pratensis* genomes, suggesting conservation in the *Lolium/Festuca* complex (bootstrap support, 100; Knorst et al., 2019; Samy et al., 2020). A single ortholog of *CO9/Ghd7* was also present on scaffold_10876 which was also present in the *L. multiflorum* and *F. pratensis* genomes (bootstrap support, 100).

Next, we aligned the CCT domains of *LpVRN2a*, *LpVRN2b*, and *LpCO9* alongside homologs from wheat, barley, *B. distachyon*, *L. multiflorum*, *F. pratensis* and rice to determine whether any known functional residues were mutated in ryegrass species. Arginine residues 16, 35 and 39 have been reported to be mutated in non-functional versions of ZCCT in spring wheat, and these residues were unchanged in *L. perenne* or *L. multiflorum*, suggesting that the CCT domains of these proteins were likely to be functional (Distelfeld et al., 2009; Supplementary Figure 3A). In addition, residues conserved amongst a wide range of CCT-domain proteins, identified by Cockram et al. (2012), were unchanged in *L. perenne*, *L. multiflorum* and *F. pratensis* VRN2 and CO9-like proteins (Supplementary Figure 3A).

We also examined whether these three putative repressors were syntenic to known *VRN2* genes in other grasses. Only *LpVRN2a* was present on a scaffold with more than one annotated gene, allowing us to easily determine syntenic regions from *B. distachyon* and wheat. The genes surrounding *LpVRN2a* were present on chromosome 1 from *B. distachyon* and 4B from durum wheat (*Triticum turgidum*; Supplementary Figure 3B). *LpVRN2a* was downstream of a norcoclaurine synthase gene; however, it was absent from *B. distachyon* and wheat. This suggests that *LpVRN2a* likely results from a novel insertion of a *VRN2-like* gene during the divergence of the *Lolium* genus.

### Expression Analysis Indicates *LpVRN2a, LpVRN2b* and *LpCO9* May Function as Floral Repressors

To gain a better understanding of the likely roles of *LpVRN2a, LpVRN2b* and *LpCO9*, we examined their expression pattern during vernalization in previously published RNAseq datasets from two ecotypes with differing vernalization requirements (Paina et al., 2014). *LpVRN2a* showed increased expression during vernalization in Falster compared to pre-vernalization levels, but was very lowly expressed in Veyo, while *LpVRN2b* showed very low expression in both varieties (Figures 2B,C). As the experiment had been conducted solely in short days, it is possible that both *VRN* homologs are repressed before cold treatment in these samples, as *VRN2-like* genes are repressed in short days in other species (Dubcovsky et al., 2006). *LpCO9* was repressed during vernalization and stayed repressed on introduction to LD, similar to *VRN2* from wheat as previously reported (Figure 2D; Paina et al., 2014).

**FIGURE 3:**
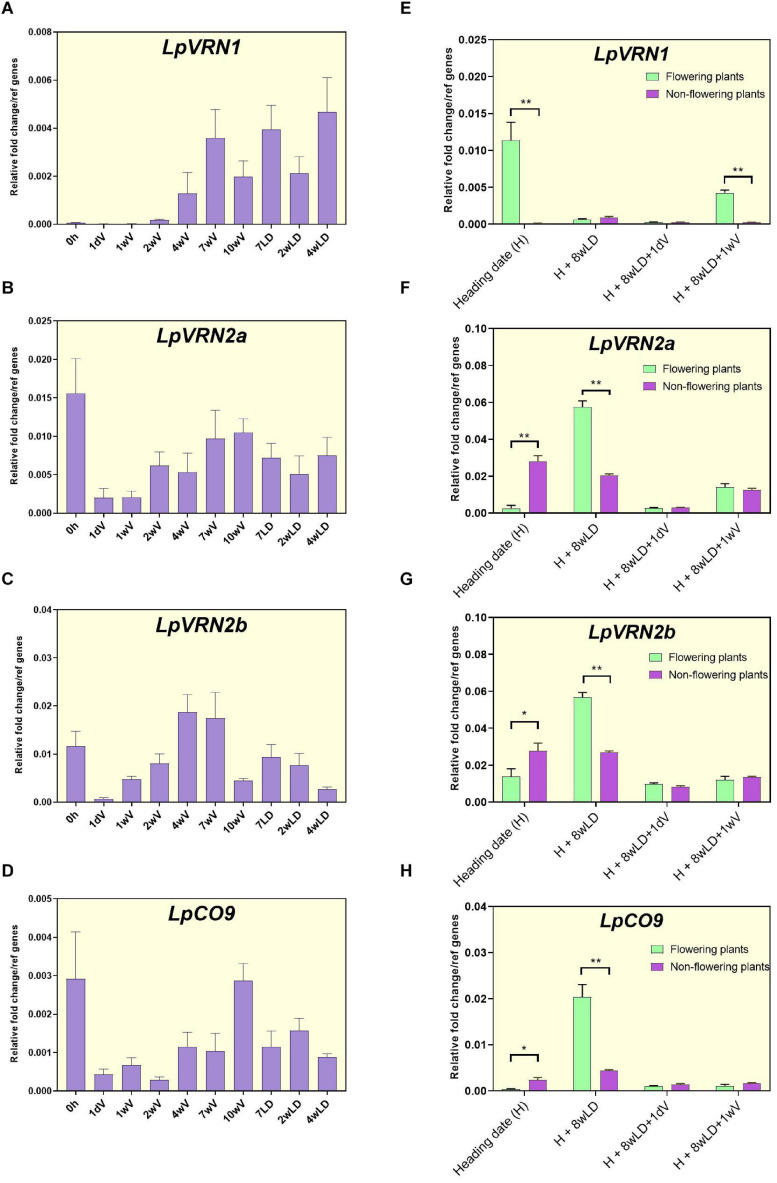
Expression patterns of *VRN1* and *VRN2-like* genes from *L. perenne* during vernalization, heading, and re-vernalization. **(A–D)** Expression pattern of *LpVRN1*, *LpVRN2a*, *LpVRN2b*, and *LpCO9* during and after vernalization using qPCR in seedlings of the ONE50 cultivar. Expression was normalized to the geometric mean of two reference genes (*ExP* and *THP*) and shows the mean and standard deviation of two biological replicates of leaf tissue from ∼10 pooled seedlings. **(E–H)** Expression pattern of *LpVRN1*, *LpVRN2a*, *LpVRN2b*, and *LpCO9* at heading **(H)**, 8 weeks after heading (H + 8wLD), and during reintroduction to vernalization conditions (H + 8wLD + 1dSDV and H + 8wLD + 1wSDV). Data is shown for plants that flowered (*n* = 3) and plants that did not flower (*n* = 3). Expression was normalized to the geometric mean of two reference genes (*ExP* and *THP*), the mean and standard deviation of three biological replicates is shown. Each biological replicate is from several leaf samples from a single plant. Asterisks represent the results of Student’s *t*-test between flowering and non-flowering results (*p < 0.1; **p < 0.05). LD, long days; SDV, short days with vernalization.

We further characterized these expression patterns in the ONE50 cultivar, alongside the expression of *LpVRN1*, homologs of which act as repressors of *VRN2* (Chen and Dubcovsky, 2012; Deng et al., 2015; Figure 3). *LpVRN1* increased expression during vernalization, as previously demonstrated (Andersen et al., 2006; Figure 3A). *LpVRN2a* showed a similar expression pattern to the RNAseq data with a peak after several weeks of vernalization (Figure 3B). In contrast to the RNAseq data, *LpVRN2b* was expressed in ONE50 and showed a similar pattern to *LpVRN2a* (Figure 3C). *LpCO9-like* was also expressed; however, the pattern did not closely match that seen in the RNA seq data (Figure 3D). All three of these genes showed a reduction upon transfer from long days to vernalization conditions but unexpectedly increased later during vernalization, in contrast to similar experiments in wheat and barley (Figures 3B–D).

Samples used for qPCR time-course experiments were pooled from a number of related, but not identical ONE50 seedlings. Upon introduction to long days in our conditions, certain plants did not undergo flowering, suggesting that the pooled samples may contain induced and un-induced seedlings. We reasoned that the increase in expression of *LpVRN2a*, *LpVRN2b* and *LpCO9* seen after ∼4 weeks of vernalization was due to expression in seedlings that were not responding to the vernalization treatment. To test this, we performed qPCR on flowering and non-flowering plants derived from this experiment (Figures 3E–H). As expected, *VRN1* was highly expressed in flowering plants at heading (H), but not in non-flowering plants (Figure 3E). *LpVRN2a*, *LpVRN2b* and *LpCO9* expression was low in flowering plants and high in non-flowering plants at heading (H; Figures 3F–H), suggesting that non-flowering seedlings may be the source of the observed increase in gene expression of these genes after ∼4 weeks of vernalization (Figures 3B–D).

As ryegrass is perennial, the vernalization response must be reset after flowering. We hypothesized that this would entail reversion of *LpVRN1* and the floral repressors to their pre-vernalization levels, and that they would be able to respond to vernalization conditions again. We re-tested gene expression in the flowering and non-flowering plants from above 8 weeks after the completion of flowering (H + 8wLD), and also after 1 day (+ 1dV) and 1 week (+ 1wV) in vernalization conditions. Eight weeks after heading, *LpVRN1* expression had returned to low pre-vernalization levels (Figure 3E), while *LpVRN2a, LpVRN2b* and *LpCO9* had increased in plants that had previously flowered (Figures 3F–H). Upon re-introduction to vernalization conditions, *LpVRN1* and the floral repressors responded as expected, suggesting that the plants were competent to undergo vernalization again (Figures 3E–H). Surprisingly, plants that had not initially flowered showed lower expression of floral repressors 8 weeks after heading (H + 8wLD) than plants that had flowered (Figures 3F–H). However, *LpVRN2a, LpVRN2b* and *LpCO9* expression was still repressed upon introduction to vernalization conditions in these plants, suggesting that they were competent to respond to vernalization despite their already low levels of expression (Figures 3F–H). Interestingly, *LpVRN1* did not strongly respond to vernalization conditions in the non-flowering plants, perhaps indicating that some genetic differences underlay the initial non-flowering phenotype (Figure 3E). In parallel, we assayed clones of these plants under short days (without cold; Supplementary Figure 4). *LpVRN1* did not respond to short days, while the repressors responded in a similar manner in short days as they did to short days with cold (Supplementary Figure 4).

### Variation in the Expression of Key Flowering-Time Genes Occurs in Ryegrass Genotypes With Different Vernalization Responses

Given the differences in *LpVRN1* response seen in ONE50 plants above, we next aimed to determine whether the expression levels of *LpVRN1*, *LpVRN2a*, *LpVRN2b* and *LpCO9* might contribute to the differences in vernalization response in different ryegrass genotypes (Table 1). Ryegrass genotypes were selected from a range of geographic locations, as well as a number of commercially grown cultivars with different flowering habits, in an effort to capture a wide array of genetic variation (Table 1; Faville et al., 2020). In wheat and barley, vernalization induces changes in gene expression of *VRN1*, firstly transiently, and then through stable epigenetic marks (Yan et al., 2003; Oliver et al., 2009; Diallo et al., 2012). Many spring varieties of wheat, as well as some ryegrass genotypes have high levels of *VRN1* expression prior to vernalization which contributes to their lack of vernalization requirement (Andersen et al., 2006; Chu et al., 2011). We were interested in detecting variation in the level of *LpVRN1* prior to vernalization and the transient response which may be indicative of genetic variation underlying different flowering habits. Overexpression of *VRN2* and *CO9-like* genes also leads to delayed flowering, and may be another mechanism by which ryegrass can modulate its vernalization response (Kikuchi et al., 2012; Woods et al., 2016). As VRN1 regulates *VRN2* expression at the promoter in other species, the absence of *VRN2* repression upon transient VRN1 induction may indicate changes at the *VRN2* promoter which could also contribute to varying vernalization responses (Deng et al., 2015). We predicted that the expression levels of *LpVRN1* may be higher in earlier flowering varieties [similar to that seen in Veyo (early) compared to Falster (late); Andersen et al., 2006], and that the expression levels of the repressors may be lower. Alternatively, response to vernalization conditions may occur sooner in earlier flowering varieties, which would be illustrated by large changes in expression upon introduction to vernalization conditions. Genotypes with unusual expression levels or responses may contain useful genetic variation in the assayed genes that can be investigated further.

We examined the expression level of *LpVRN1*, *LpVRN2a*, *LpVRN2b* and *LpCO9* prior to introduction to vernalization conditions in pooled 3-week-old seedlings to determine the pre-vernalization level of each gene (Supplementary Figure 5). To determine whether there was variation in the initial/transient response of these genes to vernalization conditions we also tested the expression of these genes after 2 days in cold (4°C) and dark and compared the values (Supplementary Figure 5). Gene expression was detected for all genes in all genotypes, except for *LpCO9* in the Medea genotype (Supplementary Figure 5). In terms of their transient response to vernalization conditions, all plants had higher *LpVRN1* expression after 2 days of cold, exemplified most clearly in the Iranian ecotype (Supplementary Figure 5E). *LpVRN2a* and *LpVRN2b* were repressed in all genotypes except Medea and Barberia, while *LpCO9* varied greatly between genotypes upon transfer to vernalization conditions (Supplementary Figure 5F–H). Despite our earlier predictions, there was no association between early flowering and high *LpVRN1* or low levels of repressors, and the transient response also did not appear to be significantly associated with flowering habit (Supplementary Figure 6). However, *LpCO9* was more highly expressed in late-flowering genotypes prior to vernalization, which may implicate it in the late-flowering phenotype (Supplementary Figure 6).

**FIGURE 4:**
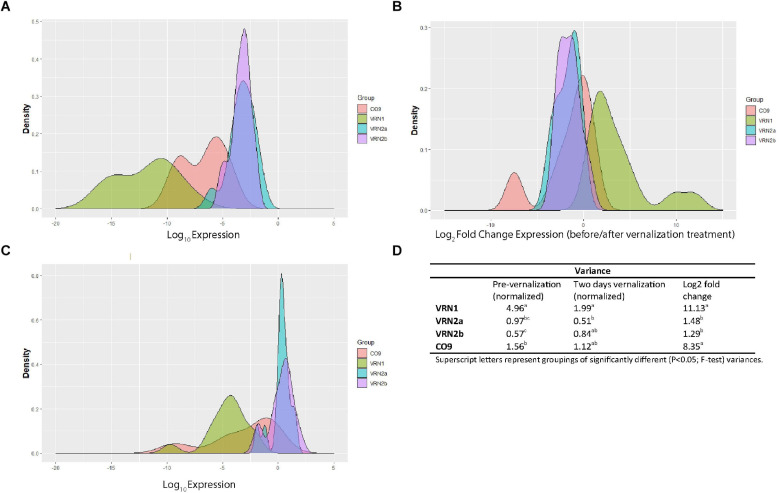
Analysis of the variability in expression and response to vernalization treatment of *LpVRN1, LpVRN2a, LpVRN2b* and *LpCO9* in a variety of different ryegrass genotypes. **(A)** Density plot of log-transformed gene expression before transfer to vernalization conditions. **(B)** Density plot of log-transformed gene expression after 2 days in vernalization conditions. **(C)** Density plot of log_2_ fold change in gene expression after transfer to vernalization conditions. **(D)** Table showing significant differences in variances (*p* < 0.05; F-test) between each gene. *LpVRN1* and *LpCO9* were more variable than *LpVRN2a* and *LpVRN2b*.

Although we did not detect strong associations between flowering habit and gene expression in most cases, there was a high level of variation in expression and response between genotypes, particularly in *LpVRN1* and *LpCO9* which may indicate the presence of natural variation. We examined the variance between genotypes of *LpVRN1*, *LpVRN2a*/*b* and *LpCO9* expression before and after 2 days in vernalization conditions, and also the variance in response (expressed as log_2_ fold change between conditions) to determine which genes are most likely to have underlying genetic variation (Figure 4). *LpVRN1* and *LpCO9* were highly variable, both in terms of expression level, and in terms of response, while *LpVRN2a* and *LpVRN2b* were significantly less variable (Figure 4). Overall, these results suggest that *LpCO9* might hold more natural variation in expression than *LpVRN2a* and *LpVRN2b*. Natural variation in *LpVRN1* has already been demonstrated and may exist in the genotypes examined here as well (Andersen et al., 2006).

## Discussion

Ryegrass flowering impacts metabolizable energy and seed production, and is therefore an important trait for breeders to target. The advent of genomic selection now allows breeders to more accurately select for heading date (Hayes et al., 2013). However, understanding the genes and alleles responsible for variation in heading date is required to more easily identify useful germplasm, and for more targeted approaches, such as gene editing with CRISPR/Cas9. Translating knowledge from model species and well-studied crops is therefore an efficient way to further our understanding of heading date in ryegrass.

Here, we have used available genomic resources to compile a list of likely orthologs of important flowering-time genes from *B. distachyon* in *L. perenne*. We have further confirmed orthology for *FT-like* and *VRN2-like* genes using phylogeny (Figures 1, 2). Further phylogenetic analyses may confirm the orthology between other genes identified here (e.g., MADS-box domain genes, such as *SOC1*), and additional genomic sequences may enable the identification of more genes/paralogs in ryegrass. It is hoped that this thorough analysis can be used as a reference, particularly for determining the functions of *FT-like* and *VRN2-like* genes in ryegrass. Our phylogenetic analysis of *FT-like* genes in *L. perenne* was more complete than previous analyses (Arojju et al., 2016), and incorporated expression data to strengthen hypotheses regarding the function of each *L. perenne FT-like* gene. For example, it has been suggested that the *FT-like* gene present on scaffold_13332 (*LpFT02*) corresponds to the *LpVRN3* marker (Arojju et al., 2016). However, the analysis by Arojju et al. (2016) did not include *LpFT3* (likely because it was not included in the transcriptome), which perfectly matches the entire sequence of the *LpVRN3* marker, and is supported to be the functional *FT* homolog in this (and other) studies (Skøt et al., 2011; Studer et al., 2012). Similarly, the gene associated with the vrn2_2 marker on scaffold_1808 matches more closely with *CO-like 14* from *B. distachyon* (Andersen et al., 2006; Supplementary Figure 3). The two *VRN2* homologs identified here are the closest match to *VRN2* from *B. distachyon* and also ZCCT1 and ZCCT2 from wheat (Figure 3A). In addition, no closer genes were identified in *F. pratensis* or *L. multiflorum*, suggesting that it is unlikely that more *VRN2-like* genes exist in *L. perenne* than the two identified here.

In wheat, *VRN2* is a key determinant of flowering habit between different cultivars. QTL analyses on ryegrass vernalization response identified *LpVRN1* and *LpCO* as important determinants of the different responses between Falster and Veyo cultivars (Jensen et al., 2005; Andersen et al., 2006). *LpVRN2a* and *LpVRN2b* were not identified in this screen, although the genes underlying all QTL from this experiment are yet to be identified. *LpVRN2a* has been assigned to linkage group 4 and is included in a QTL relating to winter survival (Paina et al., 2016). *LpVRN2b* and *LpCO9* have not been assigned to a linkage group in GenomeZipper or the recent high-density mapping population, making it difficult to determine whether these genes may correspond to a flowering QTL (Byrne et al., 2015; Velmurugan et al., 2016). Our data suggest that less variation in the expression levels (and response to vernalization conditions) of *LpVRN2a* and *LpVRN2b* exists in comparison to *LpVRN1* and *LpCO9* (Figure 4), which may explain why these genes have not been detected in previous QTL analyses; mutational studies or targeted F2 populations (e.g., utilizing Medea or Barberia individuals as parents, which lacked response in *VRN2a and VRN2b*, respectively) would be required to determine their function (Supplementary Figure 5). There may also be variation in *LpVRN2a* and *LpVRN2b* expression after long-term cold exposure/vernalization which was not represented in the initial response.

The high variability of *LpCO9* response (Figure 4), and high expression levels of *LpCO9* in late-flowering genotypes (Supplementary Figure 6), suggests that some genetic variation exists in the promoter of *LpCO9*, or in proteins that regulate its expression. How much variation in the expression levels of these genes exists between individuals of a cultivar/ecotype is also of interest – as we have pooled samples for our expression analysis (Supplementary Figure 5), it is possible that some individuals from each pool largely dictate the observed expression level of a gene. Whether the variability in *LpCO9* expression observed after 2 days in vernalization conditions translates to variability at later stages remains to be seen.

*VRN2* was also reported to be absent from the *L. multiflorum* transcriptome prior to vernalization, however, we have detected *LpVRN2a* and *LpVRN2b* transcripts in annual ryegrasses (Tabu, Barberia, and the Cypriot and Iranian ecotypes; Supplementary Figure 5; Czaban et al., 2015). This discrepancy may be due to differences in genotype, or to plant age, as we have used 3-week-old seedlings, compared to mature plants, which may indicate a role for *LpVRN2a* and *LpVRN2b* in repressing flowering in young annual ryegrass plants. The alleles present in *L. multiflorum* appear to have functional CCT domains (Supplementary Figure 3A), and the sequence of *L. multiflorum VRN2a* is present in the Falster transcriptome (data not shown; Paina et al., 2014). Alleles of *LpVRN2a* and *LpVRN2b* may play a more minor role in heading date and vernalization requirement compared to the large effect of different *LpVRN1* alleles. We did not detect an association between early flowering and high *LpVRN1* expression prior to vernalization (Supplementary Figure 5A), perhaps indicating that the early flowering (and annual) genotypes examined here contain novel variation affecting this trait, distinct from that which affects *LpVRN1* expression (as seen in Falster and Veyo; Andersen et al., 2006). Alternatively, *LpVRN1* expression may only be induced later in these genotypes, again suggesting that plant age plays a role in modulating expression of *VRN* genes in ryegrass.

How LpVRN1 impacts the expression level of *LpVRN2a, LpVRN2b* and *LpCO9* in ryegrass remains to be determined. In the non-flowering ryegrass plants, and in ryegrass exposed to short days in the absence of cold, *LpVRN1* was not induced, however, *LpVRN2a/b* and *LpCO9* expression was reduced in these conditions, suggesting that regulators other than LpVRN1 are able to repress these genes (Figure 3 and Supplementary Figure 3). The alleles underlying *LpVRN1*, *LpVRN2a/b* and *LpCO9* expression differences in the various genotypes we assayed here may also be of interest (Figure 4 and Supplementary Figure 5). Transgenic studies to test the function of *LpFT08*, *LpVRN2a/b* and *LpCO9* will be critical to further our understanding of these genes in *L. perenne*.

## Data Availability Statement

The datasets presented in this study can be found in online repositories. The names of the repository/repositories and accession number(s) can be found in the article/Supplementary Material.

## Author Contributions

RH, S, LB, and RM designed the experiments and wrote and edited the manuscript. RH and S performed the experiments. All authors contributed to the article and approved the submitted version.

## Conflict of Interest

The authors declare that the research was conducted in the absence of any commercial or financial relationships that could be construed as a potential conflict of interest.

## Footnotes

1 https://phytozome.jgi.doe.gov/

2 http://rice.plantbiology.msu.edu/
