# Supplementary material for: Identification and Characterization of Perennial Ryegrass (Lolium perenne) Vernalization Genes
Source: Front Plant Sci. 2021 Mar 5;12:640324. doi: 10.3389/fpls.2021.640324 (PMC7973463; doi:10.3389/fpls.2021.640324)
Supplement: Supplementary file 1 [file Table_1.DOCX]

**Supplementary Table S1.** Primers used for qPCR

| Primer name | Sequence |
| --- | --- |
| LpVRN1_qPCR_F | CGCAACCGACTCATGTATGG |
| LpVRN1_qPCR_R | TCTCAACCTTCGCCTTCAGT |
| LpVRN2a_qPCR_F | AGACCACCCACCATCATGC |
| LpVRN2a_qPCR_R | TGCTTCTCTCTCGTGCATTG |
| LpVRN2b_qPCR_F | TGAGGTGATAATGGCTATCGATG |
| LpVRN2b_qPCR_R | CCTCTTCTCCCTATACCTCATCA |
| LpFT3_qPCR_F | CCTTCTACACACTCGTGATGG |
| LpFT3_qPCR_R | GAGGGCTCTCGTAGCACATC |
| LpFT08_qPCR_F | GTACTTGCACTGGATGGTGG |
| LpFT08_qPCR_R | TGCTGGAGCAGCACAAATAC |
| LpCO9_qPCR_F | TCGTTTGCCGAATACCAGCC |
| LpCO9_qPCR_R | ACCGAACCACTCACGATTGT |
