# Supplementary figures and images for: Identification and Characterization of Perennial Ryegrass (Lolium perenne) Vernalization Genes
Source: Front Plant Sci. 2021 Mar 5;12:640324. doi: 10.3389/fpls.2021.640324 (PMC7973463; doi:10.3389/fpls.2021.640324)

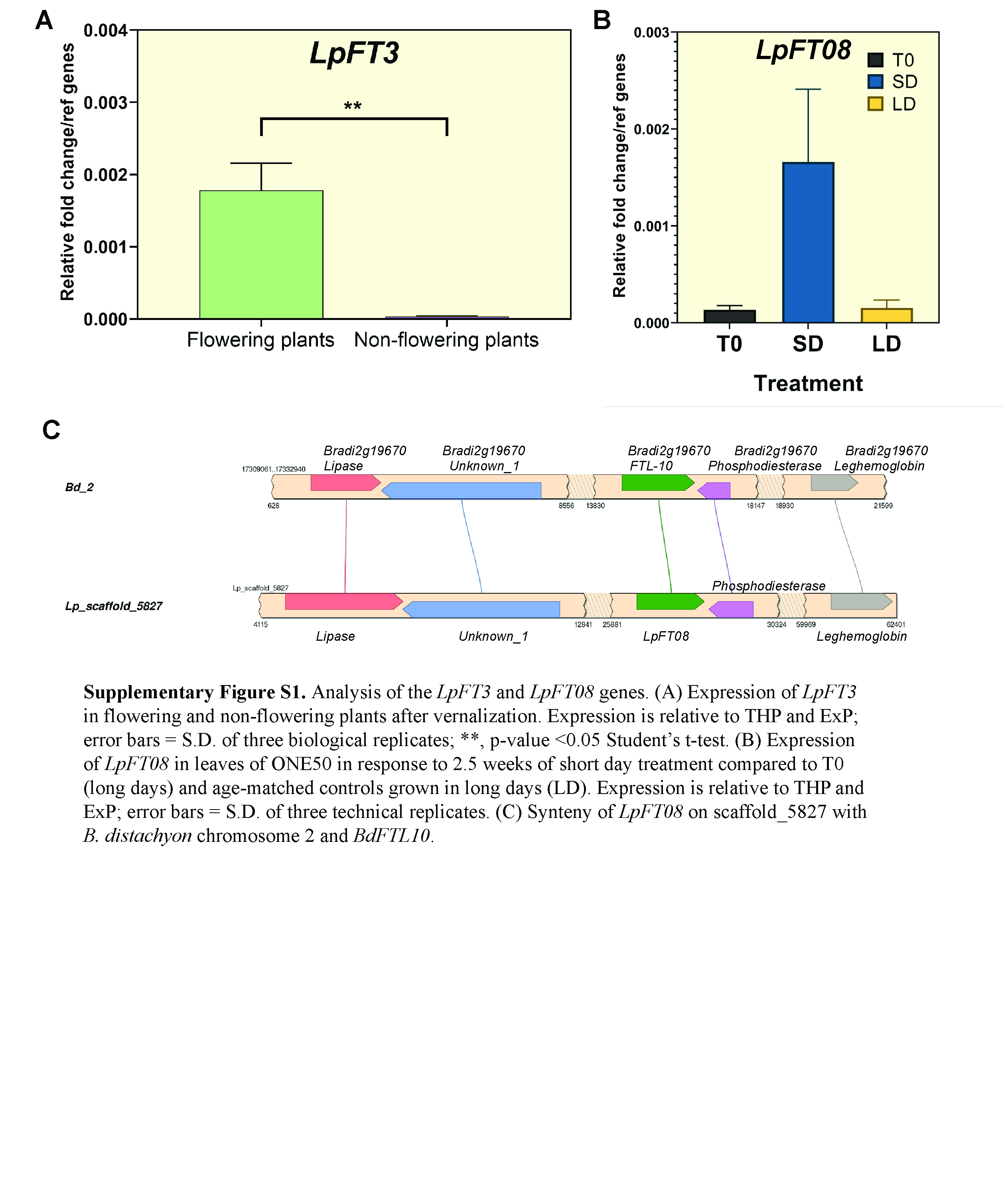

Supplement: Supplementary file 7 [file Image_1.jpg]

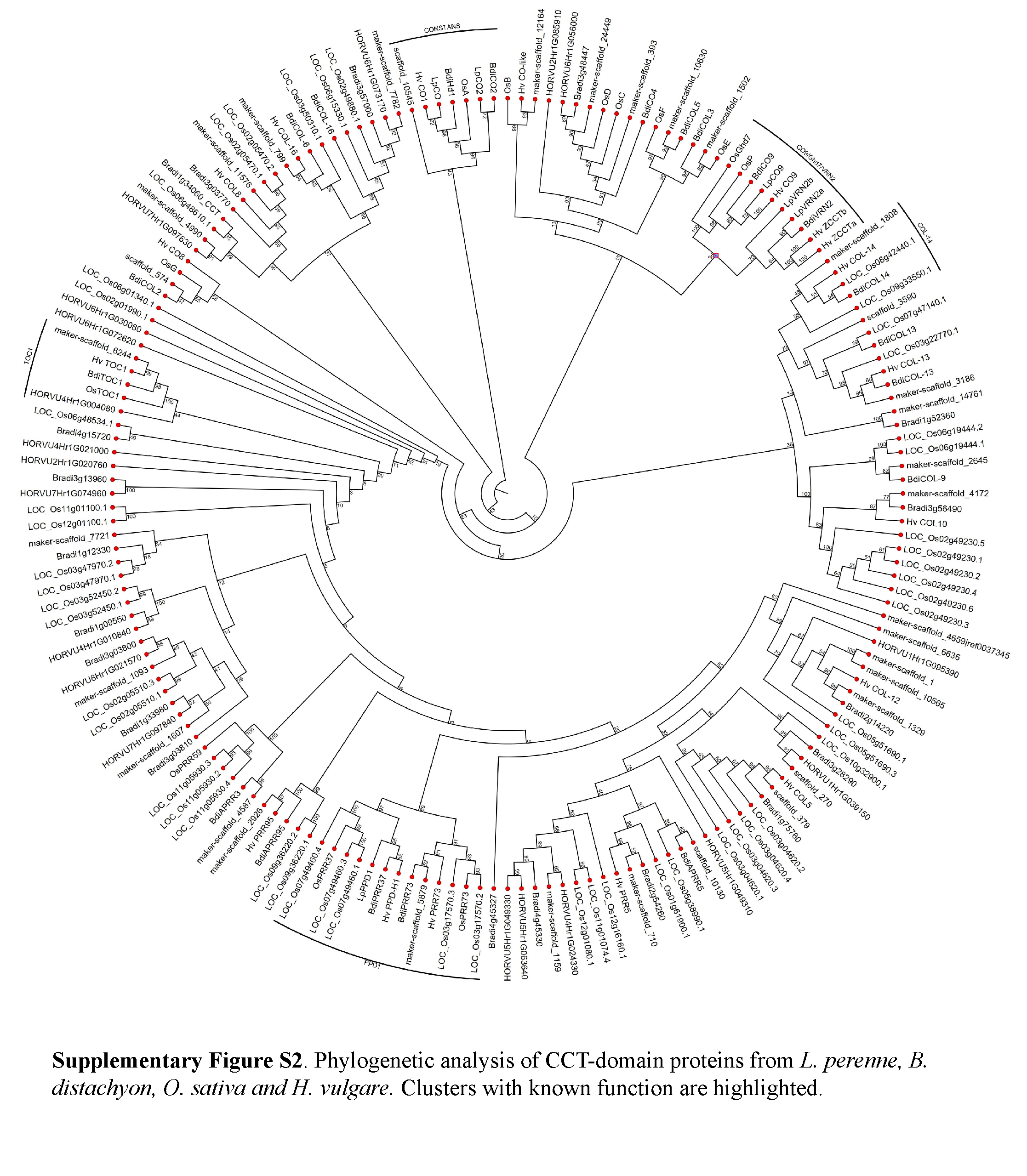

Supplement: Supplementary file 8 [file Image_2.JPEG]

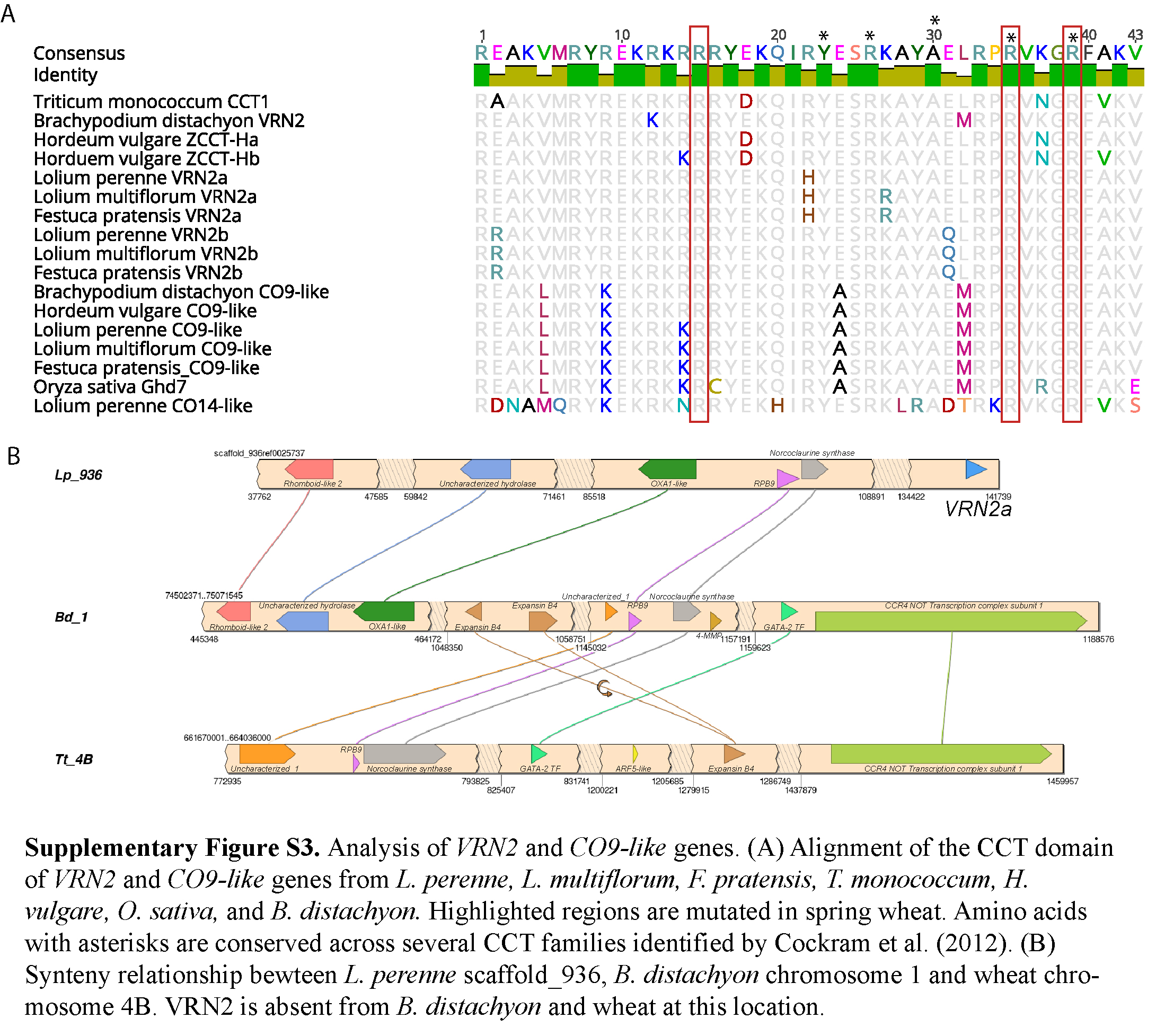

Supplement: Supplementary file 9 [file Image_3.JPEG]

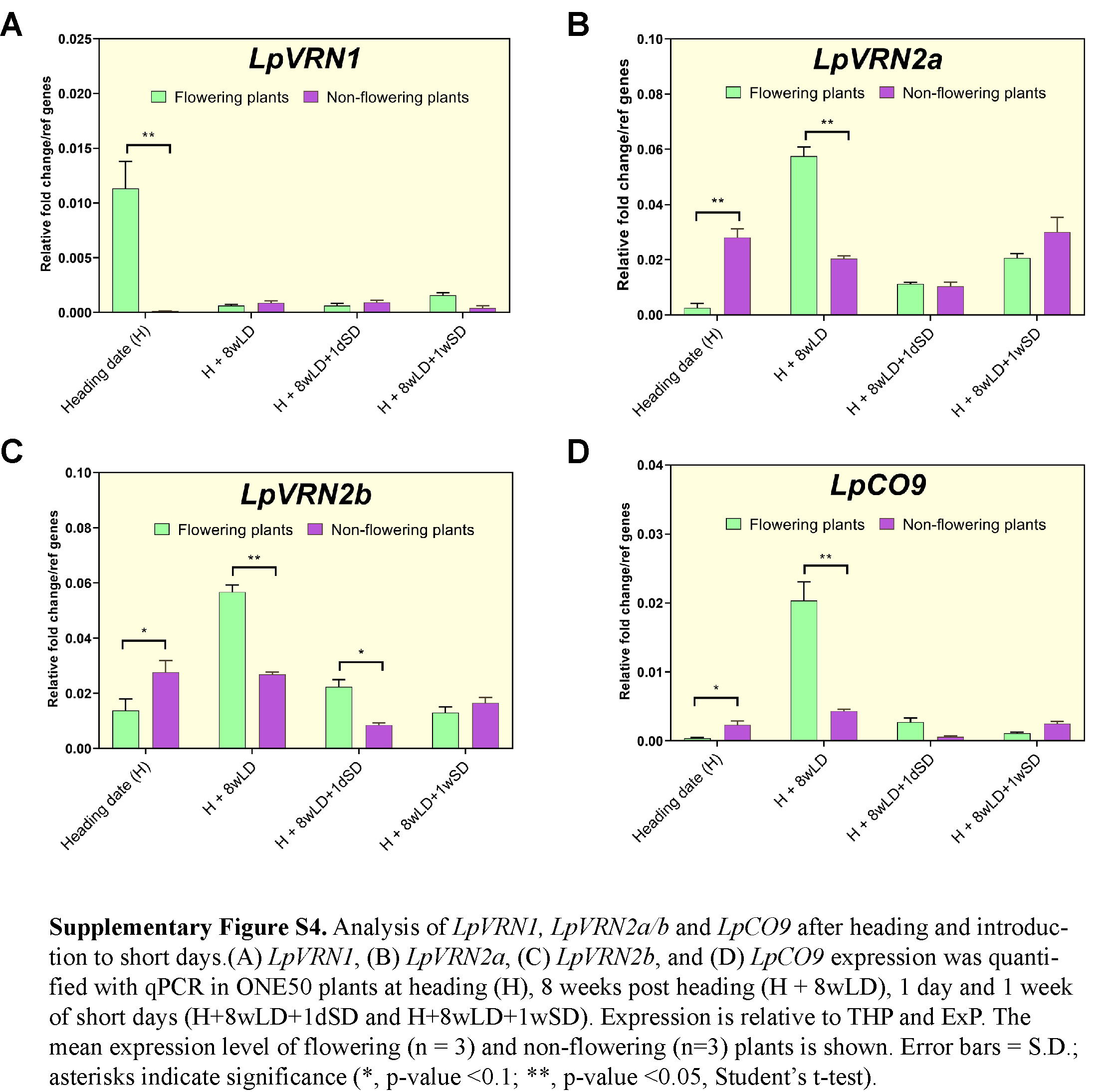

Supplement: Supplementary file 10 [file Image_4.JPEG]

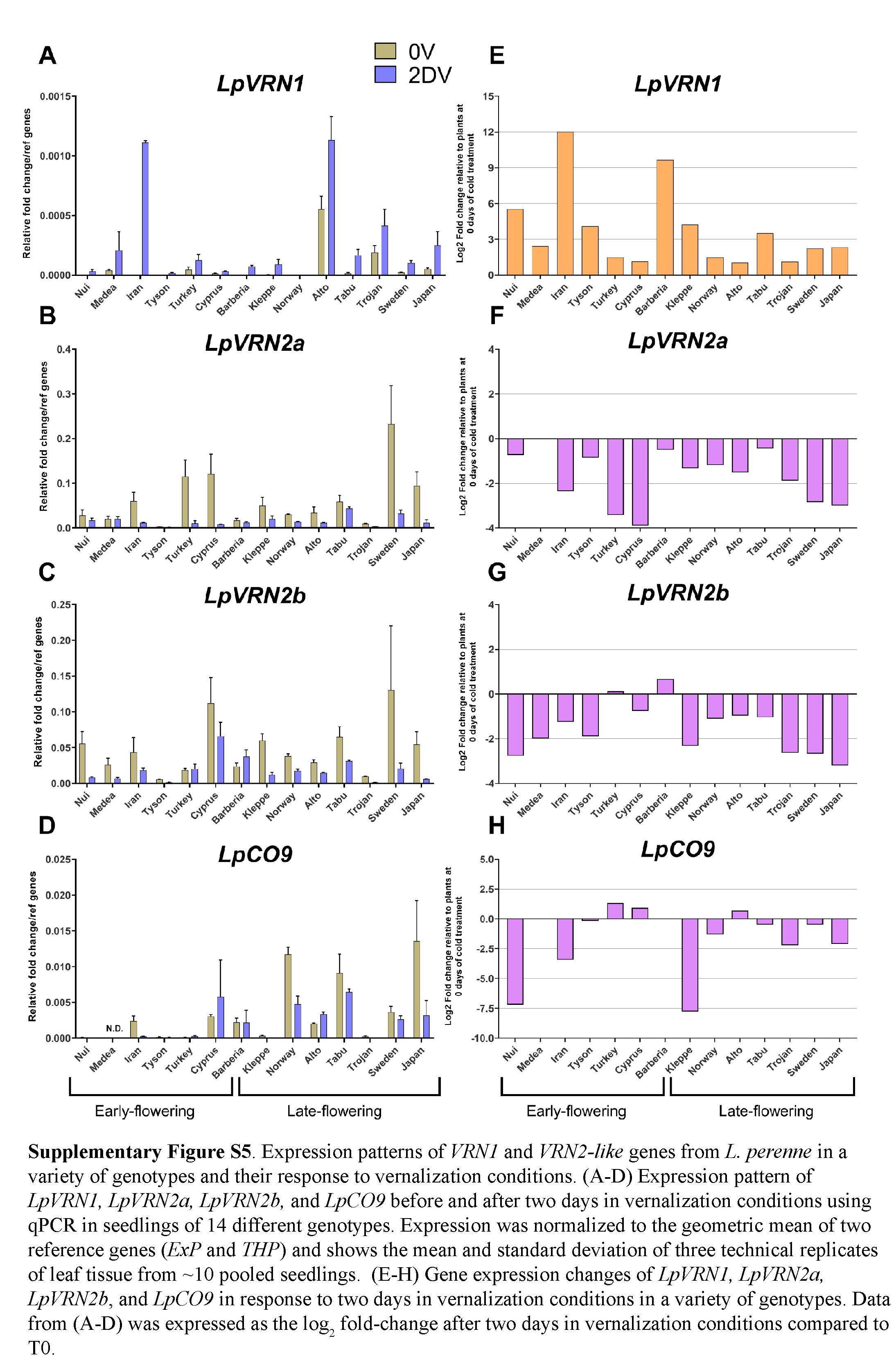

Supplement: Supplementary file 11 [file Image_5.JPEG]

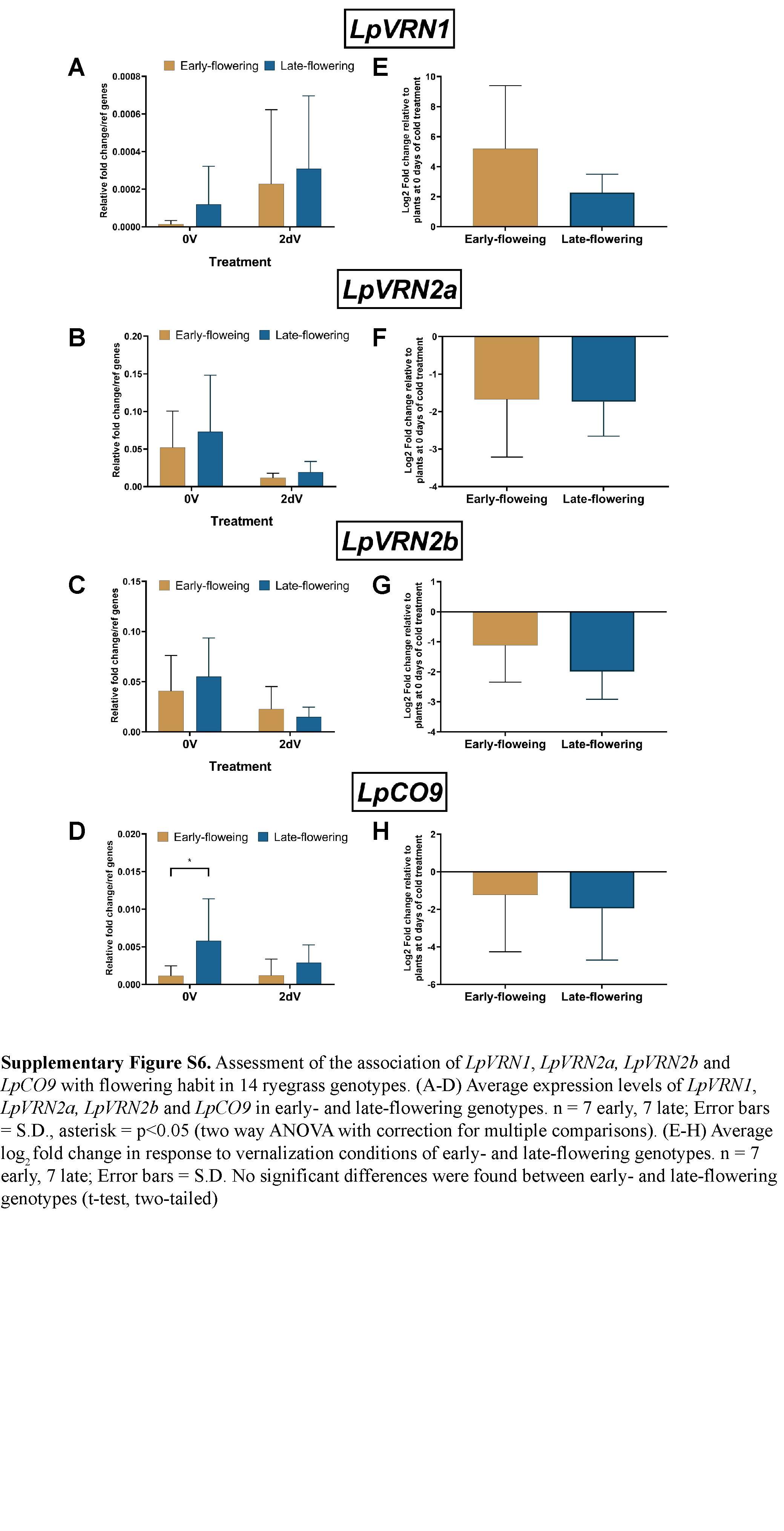

Supplement: Supplementary file 12 [file Image_6.JPEG]
